# DC‐CIK cells derived from ovarian cancer patient menstrual blood activate the TNFR1‐ASK1‐AIP1 pathway to kill autologous ovarian cancer stem cells

**DOI:** 10.1111/jcmm.13611

**Published:** 2018-03-22

**Authors:** Wenxing Qin, Ying Xiong, Juan Chen, Yongyi Huang, Te Liu

**Affiliations:** ^1^ Department of Clinical Oncology Changzheng Hospital Second Military Medical University Shanghai China; ^2^ Department of Gynaecology and Obstetrics Xinhua Hospital Affiliated to Shanghai Jiaotong University School of Medicine Shanghai China; ^3^ Gongli Hospital Affiliated to the Second Military Medical University in Pudong New Area of Shanghai City Shanghai China; ^4^ Shanghai Topbiox Co., Ltd Shanghai China; ^5^ Shanghai Geriatric Institute of Chinese Medicine Shanghai University of Traditional Chinese Medicine Shanghai China; ^6^ Department of Pathology Yale University School of Medicine New Haven CT USA

**Keywords:** cytokine‐induced killer cell, dendritic cell, ovarian cancer patient menstrual blood, ovarian cancer stem cells, targeted immunotherapy, TNFR1‐ASK1‐AIP1 pathway

## Abstract

Ovarian cancer stem cells (OCSCs) are highly carcinogenic and have very strong resistance to traditional chemotherapeutic drugs; therefore, they are an important factor in ovarian cancer metastasis and recurrence. It has been reported that dendritic cell (DC)‐cytokine‐induced killer (CIK) cells have significant killing effects on all cancer cells across many systems including the blood, digestive, respiratory, urinary and reproductive systems. However, whether DC‐CIK cells can selectively kill OCSCs is currently unclear. In this study, we collected ovarian cancer patient menstrual blood (OCPMB) samples to acquire mononuclear cells and isolated DC‐CIK cells in vitro. In addition, autologous CD44+/CD133+ OCSCs were isolated and used as target cells. The experimental results showed that when DC‐CIK cells and OCSCs were mixed and cultured in vitro at ratios of 5:1, 10:1 and 50:1, the DC‐CIK cells killed significant amounts of OCSCs, inhibited their invasion in vitro and promoted their apoptosis. The qPCR and Western blot results showed that DC‐CIK cells stimulated high expression levels and phosphorylation of TNFR1, ASK1, AIP1 and JNK in OCSCs through the release of TNF‐α. After the endogenous TNFR1 gene was knocked out in OCSCs using the CRISPR/Cas9 technology, the killing function of DC‐CIK cells on target OCSCs was significantly attenuated. The results of the analyses of clinical samples suggested that the TNFR1 expression level was negatively correlated with ovarian cancer stage and prognosis. Therefore, we innovatively confirmed that DC‐CIK cells derived from OCPMB could secret TNF‐α to activate the expression of the TNFR1‐ASK1‐AIP1‐JNK pathway in OCSCs and kill autologous OCSCs.

## 1 INTRODUCTION

Ovarian cancer is a gynaecological cancer with a very high malignancy, and this disease severely endangers the health of women. In recent years, the onset of ovarian cancer has trended towards younger patients, and the malignancy of this cancer has also increased.^1^, ^2^ Many studies in recent years have demonstrated the presence of a subpopulation of cells in ovarian cancer tissue samples that are similar to embryonic stem cells and expresses high levels of CD44, CD133 and c‐Kit (CD117) markers. These cells have the “stemness” of stem cells but also have the high proliferation, high invasiveness and high tumorigenicity features of cancer cells; therefore, these cells are known as ovarian cancer stem cells (OCSCs).^1^, ^2^, ^3^, ^4^ OCSCs have high heterogeneity and very strong resistance to traditional chemotherapeutic drugs; therefore, research and development of drugs that can be used for highly efficient and targeted killing of OCSC are particularly important.

Cytokine‐induced killer (CIK) cells refer to the CD3+/CD56+ T lymphocytes that account for only 1%‐5% of normal human peripheral blood. Currently, some studies have enriched peripheral blood mononuclear cells (PBMCs) in vitro and induced CIK cell production using cytokines intended for cancer immunotherapy.^5^, ^6^, ^7^, ^8^, ^9^, ^10^, ^11^CIK cells have 2 major heterogeneous cell subpopulations, CD3+/CD56+ and CD3+/CD8+.^5^, ^6^, ^7^, ^8^, ^9^ Some studies have indicated that CIK cells can readily recognise cancer cells and have the advantages of both the strong anti‐tumour activity of T lymphocytes and the non‐MHC‐restricted tumour‐killing ability of NK cells.^5^, ^6^, ^7^, ^8^, ^9^ Dendritic cells (DCs) are professional antigen presenting cells (APCs) that have received attention in recent years. DCs can uptake, process and present antigens to initiate T cell–mediated immune responses.^5^, ^6^, ^7^, ^8^, ^9^ DCs are the strongest known and only professional APCs that can activate resting T cells in the body and are the central link for the initiation, regulation and maintenance of immune responses.^5^, ^6^, ^7^, ^8^, ^9^ DCs are important components in tumour immunotherapy.^5^, ^6^, ^7^, ^8^, ^9^ Some studies have already confirmed that DCs combined with CIK cells (DC‐CIK) have significant killing effects on tumour cells across many systems including the blood, digestive, respiratory, urinary and reproductive systems. However, the mechanisms of action behind this phenomenon are currently still unclear.^5^, ^6^, ^7^, ^8^, ^9^, ^11^ It has been confirmed that the anti‐tumour functions of DC‐CIK cells include the following points: (1) CIK cells can recognise tumour cells through different mechanisms and release toxic granules such as granzyme/perforin to cause tumour cell lysis, thus achieving the direct tumour cell killing function. (2) CIK cells can secret many cytokines such as interferon gamma (IFN‐γ), tumour necrosis factor α (TNF‐α) and interleukin‐2 (IL‐2). These cytokines not only have direct inhibitory effects on tumour cells but also can indirectly kill tumour cells through the regulation of the immune responses in the body. (3) CIK cells express FasL (type II transmembrane glycoprotein) during the culture process to bind to Fas (type I transmembrane glycoprotein) on the cell membranes of tumour cells to induce tumour cell apoptosis. (4) DC‐CIK cells can kill many types of tumour cells and have the same potency against multidrug‐resistant tumour cells as they do against drug sensitive cells. (5) The tumour‐killing activity of DC‐CIK cells is not affected by immunosuppressive agents (such as cyclosporine and FK506), and DC‐CIK cells have very minimal toxicity against normal bone marrow haematopoietic precursor cells (less than 30%).^5^, ^6^, ^7^, ^8^, ^9^

The DOC2/DAB2 (DAB2IP) gene is localised to the 9q33.1‐q33.3 region of human chromosome 9. This gene is a tumour suppressor, and its inactivation is closely associated with tumours in the reproductive system.^12^, ^13^, ^14^, ^15^ The C2 domain of the DAB2IP protein reportedly interacts with the apoptosis signal‐regulating kinase 1 (ASK1) protein; therefore, the DAB2IP protein is also known as ASK1‐interacting protein (AIP).^12^, ^14^, ^16^, ^18^ Di Minin et al showed that when the TNF‐α‐mediated activation of the ASK1/JNK pathway was inhibited by NFκB, a mutant p53 was activated. This effect depended on the activation of the cytoplasmic DAB2IP inhibited by mutant p53.^16^ In addition, some studies have reported that the ASK1‐AIP1‐JNK/p38MAPK pathway is downstream of TNF‐α. External TNF‐α can stimulate the activation of the intracellular ASK1‐AIP1‐JNK/p38MAPK pathway to eventually induce cell apoptosis or necrosis.^19^, ^20^

Some studies have already confirmed that many types of cells are present in menstrual blood, including mononuclear cells and endometrial stem cells.^21^, ^25^ Our previous studies confirmed that transplantation of endometrial mesenchymal stem cells derived from menstrual blood could treat premature ovarian failure in mice.^22^ Furthermore, endometrial stem cells derived from menstrual blood could also be directionally induced to differentiate into various somatic cells, including neurons, osteoblasts and adipose cells, in vivo and in vitro.^21^, ^25^ Therefore, cells in menstrual blood have many potential biological functions.

Based on the above findings, we proposed a scientific hypothesis. Mononuclear cells were collected from ovarian cancer patient menstrual blood (OCPMB) in order to prepare DC‐CIK cells in vitro. Whether these cells were capable of targeted killing effects of autologous OCSCs was investigated. In addition, the relevant mechanism was studied in depth to examine whether DC‐CIK cells derived from menstrual blood exerted killing effects on OCSCs by targeting the TNFR1‐ASK1‐AIP1‐JNK pathway.

## 2 MATERIALS AND METHODS

### 2.1 Isolation and culture of tissue‐derived OCSCs

The experiment was performed according to previously described methods.^2^ Briefly, surgically isolated tissues from 6 ovarian cancer patients (Table S1) were minced, digested with 0.25% trypsin (Gibco, Gaithersburg, MD, USA) and centrifuged at 1500 r/min for 5 minutes. The cell precipitates were collected and incubated with mouse anti‐human CD133‐FITC antibodies and rabbit anti‐human CD44‐APC antibodies (e‐Bioscience Inc., San Diego, CA, USA) in vitro at 4°C for 30 minutes. Next, CD44+/CD133+ OCSCs were sorted from the sample by flow cytometry.

### 2.2 Isolation and stimulation of DC and CIK cells derived from OCPMB

A sterile one time menstrual cup (S‐Evans Biosciences) was used to collect menstrual blood. The patient was first in the aseptic ward. The aseptic menstrual cup was then removed from the sealed package. In order to ensure that menstrual cup was used under sterile condition, before collecting menstrual blood, 75% ethyl alcohol was used to wipe menstrual cup for 3 times. Then, the whole menstrual cup was put into the vagina of the patient after it was completely dried. After the menstrual blood was collected, the menstrual cup was removed in the sterile environment and collected menstrual blood in the aseptic biological safety cabinet. The experiment was performed according to previously described methods.^2^ Briefly, 10 mL of menstrual blood was collected from each of the 6 ovarian cancer patients. PBMCs were isolated from the menstrual blood sample using the Cobe Spectra Apheresis System (CaridianBCT, USA). For the induction of CIK cells, PBMCs were cultured in the serum‐free X‐VIVO 20 culture medium (Cambrex, USA) supplemented with 50 ng/mL anti‐human CD3ε antibody (e‐Bioscience), 100 U/mL recombinant human interleukin (rhIL)‐1a (Gibco) and 1000 U/mL recombinant human interferon (rhIFN)‐γ (Gibco) at 37°C and 5% CO_2_ for 24 hours; subsequently, 300 U/mL recombinant human IL‐2 (rhIL‐2, Gibco) was added. For the induction of DCs, PBMCs derived from menstrual blood were cultured in serum‐free X‐VIVO 20 culture medium supplemented with 1000 U/mL rhIL‐4 (Gibco) and 500 U/mL granulocyte/macrophage colony‐stimulating factor (GM‐CSF) (Gibco) at 37°C and 5% CO_2_ for 7 days continuously; subsequently, autologous tumour lysates (100 μg/mL) were added and co‐cultured for 24 hours to activate the DCs.

### 2.3 Extraction of total RNA and quantitative polymerase chain reaction (qPCR)

Total RNA from each group of cells was extracted using Trizol reagent according to the manufacturer's instructions. Total RNA was treated with DNase I (Sigma‐Aldrich), quantified and reverse transcribed into cDNA using the ReverTra Ace‐α First Strand cDNA Synthesis Kit (TOYOBO). Quantitative reverse transcription‐PCR (qRT‐PCR) was performed with a RealPlex4 real‐time PCR detection system from Eppendorf Co. Ltd. (Germany). SYBR Green Real‐Time PCR Master Mix (TOYOBO) was used as the fluorescent dye in the nucleic acid amplification. qRT‐PCR was completed with 40 amplification cycles as follows: denaturation at 95°C for 15 seconds, annealing at 58°C for 30 seconds and extension at 72°C for 42 seconds. The relative gene expression levels were calculated using the 2^−ΔΔCt^ method (ΔCt=Ct_genes–Ct_18sRNA; ΔΔCt=ΔCt_all_groups–ΔCt_blankcontrol_group). The mRNA expression levels were normalised to the expression level of 18s rRNA. The primers for amplification of each gene are shown in Table S2.

### 2.4 Western blot

Briefly, total proteins from the cells in each group were subjected to 12% denaturing sodium dodecyl sulphate‐polyacrylamide gel electrophoresis (SDS‐PAGE). The proteins were then transferred onto a polyvinylidene fluoride (PVDF) membrane (Millipore). The membrane was blocked, washed and incubated with primary antibodies at 37°C for 45 minutes (Table S3). After the membrane was fully washed, it was incubated with secondary antibodies at 37°C for 45 minutes (Table S3). The membrane was washed with Tris‐buffered saline/Tween 20 (TBST) 4 times at room temperature for 14 minutes each time. Next, the samples were exposed and imaged (Sigma‐Aldrich Chemical) using the enhanced chemiluminescence (ECL) method (Pierce Biotechnology).

### 2.5 Annexin V/propidium iodide (PI) staining and flow cytometry

The experiment was performed according to the instruction manual of the Annexin V‐FITC Apoptosis Detection Kit (Beyotime Biotechnology, China). Briefly, adherent cells were digested using trypsin. The cells were washed with phosphate‐buffered saline (PBS) once, centrifuged to remove residual body liquid and gently resuspended in 195 μL of Annexin V‐FITC binding solution. Next, 5 μL of Annexin V‐FITC was added, and the sample was gently mixed. Finally, 10 μL of PI staining solution was added, and the sample was gently mixed and incubated at 20°C in the dark for 30 minutes. The cells were then detected using a flow cytometer (Cytomics FC 500, BECKMAN).

### 2.6 TNFR1 knockout experiment using CRISPR/Cas9

The human TNFR1 CRISPR/Cas9 knockout (KO) plasmid was purchased from Santa Cruz Biotechnology Inc. (Santa Cruz, CA, USA). The steps were performed according to the manufacturer's instructions for the plasmid. Briefly, the above plasmid was transfected into cells using the Ultracruz^®^ transfection reagent (Santa Cruz).

### 2.7 In vivo xenograft experiments

Briefly, DC or DC‐CIK cells were mixed with OCSCs at a ratio of 10:1 and cultured in vitro for 24 hours. Then, cells were then collected and about 1 × 10^5^ cells were inoculated BALB/c nude/nude mice. Each experimental group consisted of 4 mice. After 4 months of observation, the mice were killed and tumours were stripped. The tumour was weighed and its volume was calculated according to the formula: tumour volume (mm^3^) = (*ab* ^2^)/2, where *a* represents the longest axis (mm) and *b* the shortest axis (mm). Male nude BALB/c‐nu/nu mice (6‐8 weeks old) were obtained from the Animal Research Center, Shanghai University of Traditional Chinese Medicine, China. This study was approved (Permit SHUTCMLL20140018) by the Animal Ethics Committee of Shanghai University of Traditional Chinese Medicine, in compliance with the Experimental Animal Regulations of the National Science and Technology Commission, China.

### 2.8 Statistical analysis

Each experiment was performed as least 3 times, and data are shown as the mean ± SE where applicable, and differences were evaluated using Student's *t* tests. The probability of *P* < .05 was considered to be statistically significant.

## 3 RESULTS

### 3.1 DCs and CIK cells could be induced from OCPMB‐derived mononuclear cells in vitro

**Figure 1:**
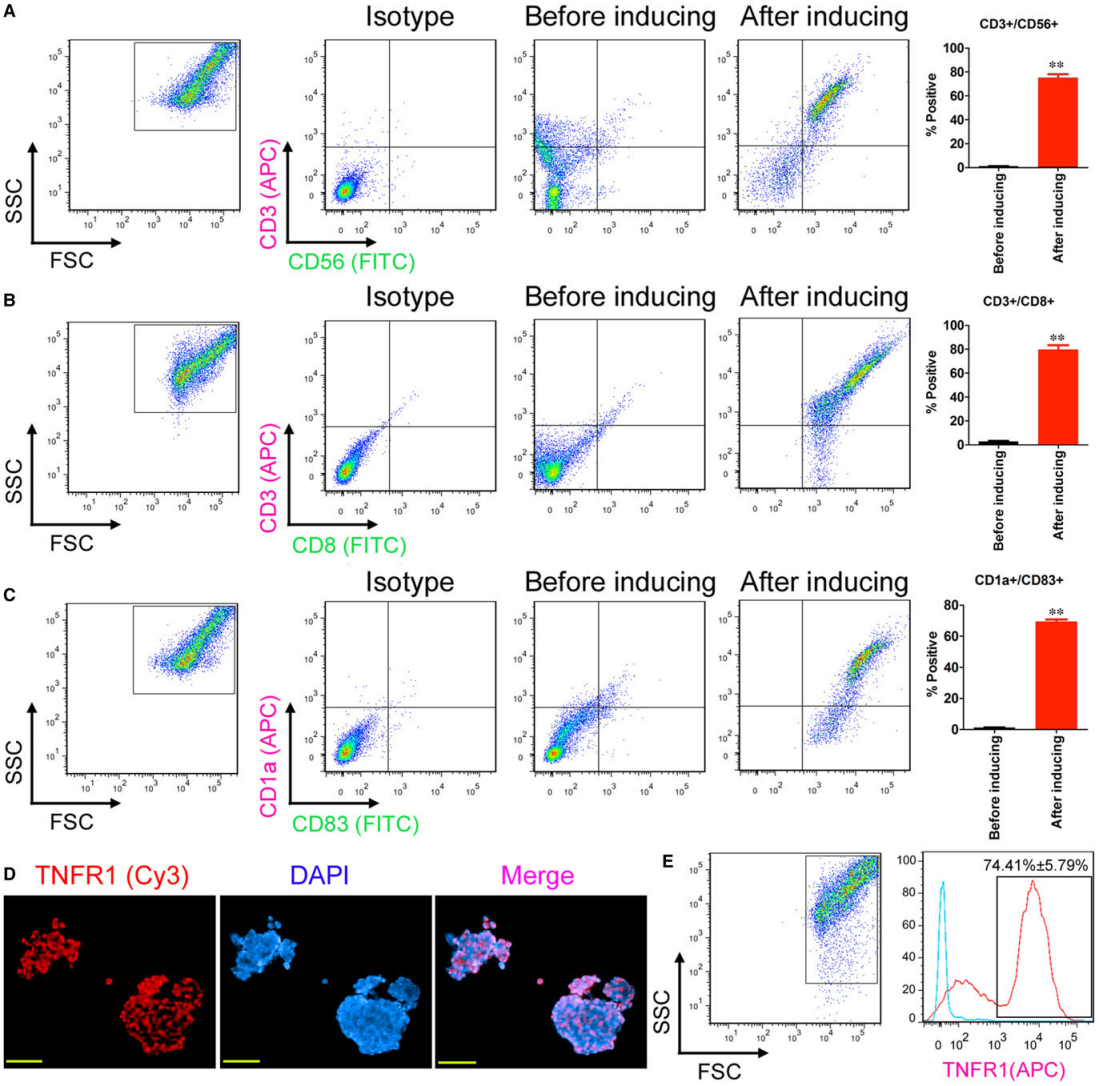
Induction of OCPMB‐derived mononuclear cells in vitro to differentiate into DC and CIK cells. A, FACS detection results showed that stimulated mononuclear cells significantly expressed high levels of the CIK markers CD3 and CD56. **P < .01 vs before induction; n = 6; B, FACS detection results showed that stimulated mononuclear cells significantly expressed high levels of the CIK makers CD3 and CD8. **P < .01 vs before induction; n = 6; C, The FACS detection results showed that the stimulated mononuclear cells significantly expressed high levels of the DC markers CD1a and CD83. **P < .01 vs before induction; n = 6; D, Immunofluorescence staining results showed that the enriched CD44+/CD133+ OCSCs expressed high levels of TNFR1 protein. Scale bar = 30 μm; E, The FACS detection results showed that the positive TNFR1 protein detection rate among the enriched CD44+/CD133+ OCSCs reached approximately 74.41%

Mononuclear cells from OCPMB were collected and stimulated by cytokines in vitro. FACS detection results showed that stimulated mononuclear cells expressed significantly high levels of CD3, CD8, CD56, CD1a and CD83(Figure 1). The results suggested that OCPMB‐derived mononuclear cells could be stimulated to differentiate into DCs and CIK cells in vitro. In addition, immunofluorescence staining results showed that the enriched CD44+/CD133+ OCSCs expressed high levels of TNFR1 (Figure 1).

### 3.2 OCPMB‐derived DC‐CIK cells significantly killed CD44+/CD133+ OCSCs

**Figure 2:**
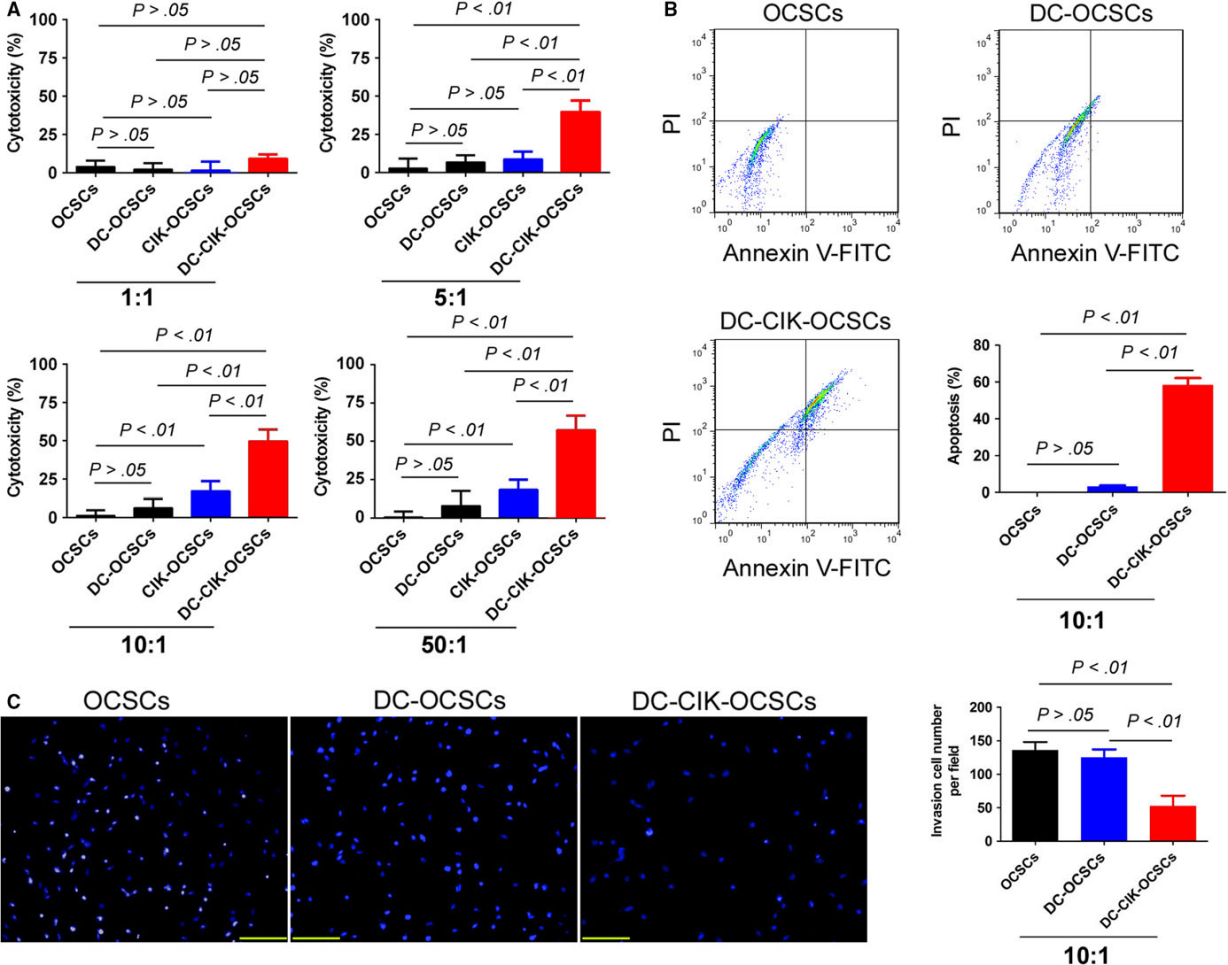
OCPMB‐derived DC‐CIK cells significantly killed CD44+/CD133+ OCSCs. A, The cytotoxicity experiment results showed that the OCSC killing rates of CIK and the OCSC killing rate of DC‐CIK cells were both significantly higher than those in the simple OCSC group and the DC‐OCSC group at the 10:1 and 50:1 ratios; B, DC‐CIK cells and OCSCs were mixed at a ratio of 10:1 and cultured in vitro for 24 h. FACS results of Annexin V/PI staining showed that the total apoptosis rate in the DC‐CIK‐OCSC group was significantly higher than in the simple OCSC group and the DC‐OSCS group; C, The transwell invasion experiment results suggested that the number of migrated cells in the DC‐CIK‐OCSC group was significantly lower than those in the simple OCSC group and the DC‐OCSC group. Scale bar = 30 μm

**Figure 3:**
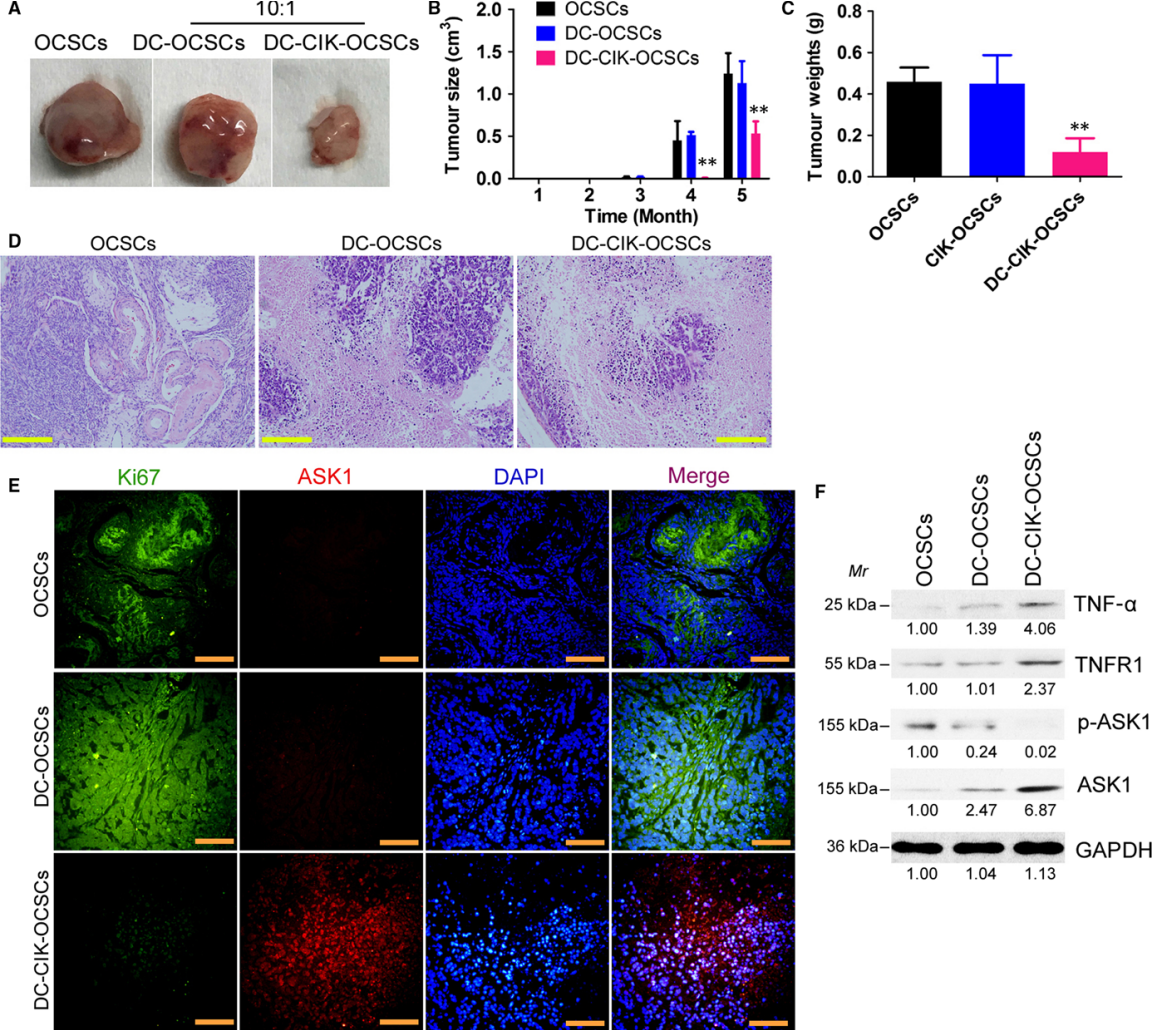
OCPMB‐derived DC‐CIK cells significantly inhibited the growth of CD44+/CD133+ OCSCs in nude mice. A, DC or DC‐CIK cells were mixed with OCSCs at a ratio of 10:1 and cultured in vitro for 24 h. The cells were collected and subcutaneously injected into nude mice. After 5 mo, tumour tissues derived from each group of cells were isolated; B, The volume of tumour tissues in the DC‐CIK‐OCSC group was significantly smaller than those in the simple OCSC group and the DC‐OCSC group. **P < .01 vs OCSCs; n = 6; C, The weight of tumour tissues in the DC‐CIK‐OCSC group was significantly lower than those in the simple OCSC group and the DC‐OCSC group. **P < .01 vs OCSCs; n = 6; D, The HE staining results showed that tumours in all groups met the characteristics of mixed‐type epithelial ovarian cancer. The tumour tissues in the DC‐CIK‐OCSC group had more necrotic cells, significantly reduced numbers of tumour cells and significantly reduced malignancy. Scale bar = 30 μm; E, The immunofluorescence staining results showed that the number of Ki67‐positive cells in tumour tissues from the DC‐CIK‐OCSC group was significantly lower than those in the other 2 groups, whereas the number of ASK1‐positive cells was significantly higher than those in the other 2 groups. Scale bar = 30 μm; F, The Western blot results suggested that the expression levels of TNF‐α, TNFR1 and ASK1 in tumour tissues in the DC‐CIK‐OCSC group were significantly higher than those in other groups, whereas the expression level of p‐ASK1 protein was significantly lower than those in other groups

DC, CIK and DC‐CIK cells were separately mixed with OCSCs at specific ratios (1:1, 5:1, 10:1 and 50:1) and cultured in vitro for 24 hours. The cytotoxicity experiment results showed that in the 10:1 and 50:1 groups, the killing rate of CIK cells on OCSCs and the killing rate of DC‐CIK cells on OCSCs were both significantly higher than those in the simple OCSC group and the DC‐OCSC group (Figure 2). The FACS results of Annexin V/PI staining showed that the total apoptosis rate in the DC‐CIK‐OCSC group was significantly higher than those in the simple OCSC group and the DC‐OCSCs group (Figure 1). In addition, transwell invasion experiment results suggested that the number of migrated cells in the DC‐CIK‐OCSC group was significantly lower than those in the simple OCSC group and the DC‐OCSC group (Figure 2). Furthermore, DC or DC‐CIK cells were mixed with OCSCs at a ratio of 10:1 and cultured in vitro for 24 hours. The cells were then collected and subcutaneously injected into nude mice. Tumour tissues were isolated after 4 months, and the measurement results showed that both the volume and weight of tumour tissues in the DC‐CIK‐OCSC group were significantly lower than those in the simple OCSC group and the DC‐OSCS group (Figure 3). The haematoxylin‐eosin (HE) staining results showed that tumours in these 3 groups all met the characteristics of mixed‐type epithelial ovarian cancer; only tumour tissues in the DC‐CIK‐OCSC group had more necrotic cells and lower malignancy (Figure 3). The immunofluorescence staining results showed that the number of Ki67‐positive cells in tumour tissues from the DC‐CIK‐OCSC group was significantly lower than those in other 2 groups, whereas the number of ASK1‐positive cells was significantly higher than those in the other 2 groups (Figure 3). In addition, Western blot results suggested that the expression levels of TNF‐α, TNFR1 and ASK1 in tumour tissues from the DC‐CIK‐OCSC group were significantly higher than those in other groups, whereas the expression level of phosphorylated ASK1 (p‐ASK1) was significantly lower than those in other groups (Figure 3). In summary, OCPMB‐derived DC‐CIK cells significantly inhibited in vitro proliferation and invasion as well as in vivo tumorigenic abilities of OCSCs.

### 3.3 OCPMB‐derived DC‐CIK cells secreted inflammatory factors

**Figure 4:**
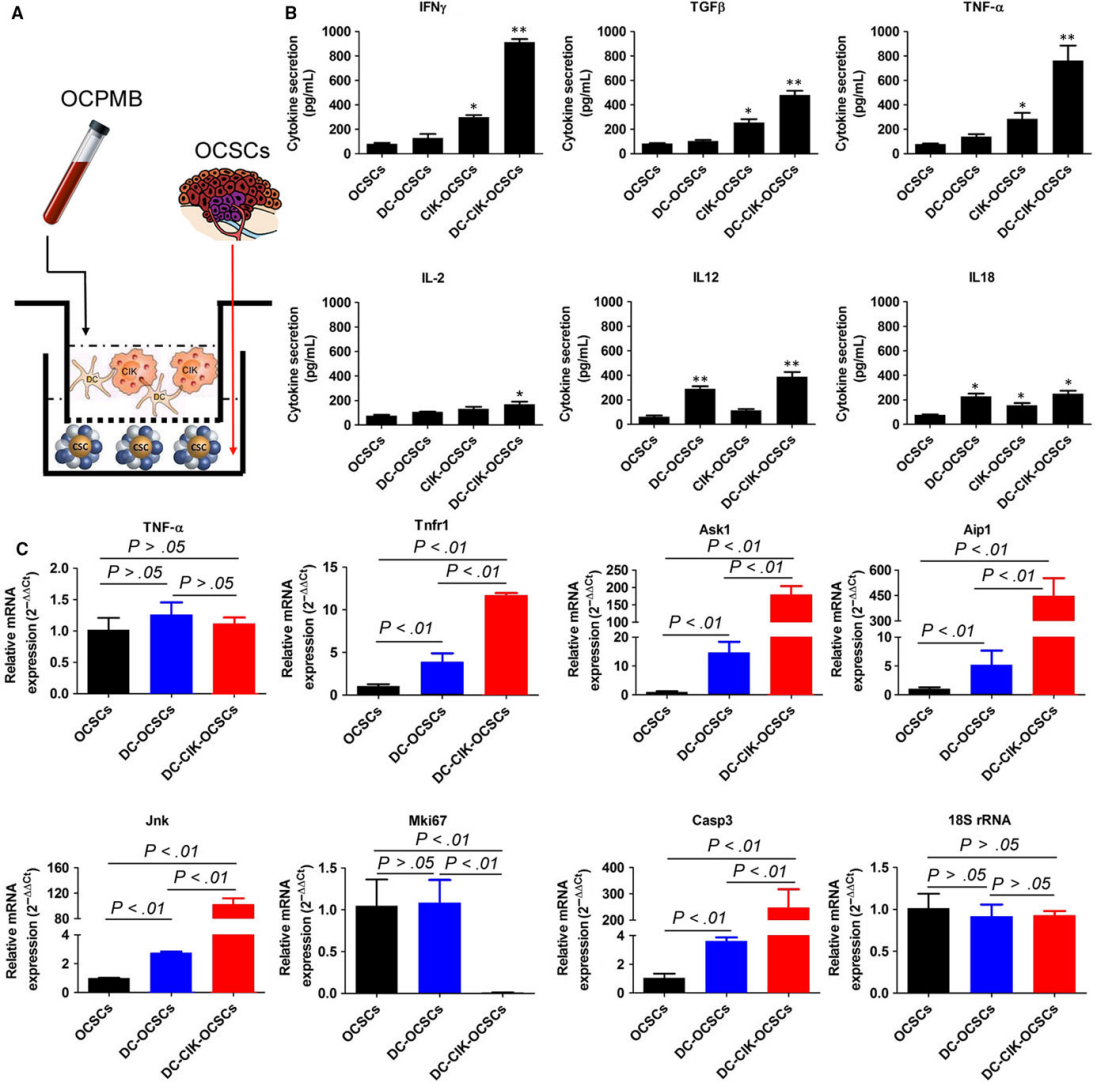
PCPMB‐derived DC‐CIK cells secreted inflammatory factors to stimulate activation of the TNFR1‐ASK1‐AIP1‐JNK pathway. A, DC, CIK and DC‐CIK cells were separately mixed with OCSCs at a ratio of 10:1 and cultured for 24 h in vitro; B, The ELISA results suggested that the levels of IFN‐γ, TGFβ and TNF‐α in the cell culture medium in the DC‐CIK‐OCSC group were significantly higher than those in the OCSC group, the simple CIK‐OCSC group and the DC‐OCSC group; C, The qPCR results showed that the mRNA expression levels of Tnfr1, Ask1, Aip1, Jnk and caspase‐3 in OCSC from the DC‐CIK‐OCSC group and the DC‐OCSC group were significantly higher than those in the simple OCSC group; however, the mRNA expression levels of the above genes in the DC‐CIK‐OCSC group were significantly higher than that in the DC‐OCSC group. The expression level of the nuclear proliferation factor mKi67 in the DC‐CIK‐OCSC group was significantly lower than that in the DC‐OCSC group and the simple OCSC group. **P < 0.01 vs OCSCs; *P < 0.05 vs OCSCs; n = 6

DC, CIK and DC‐CIK cells were separately mixed with OCSCs at a ratio of 10:1 and cultured in vitro for 24 hours (Figure 4). The enzyme‐linked immunosorbent assay (ELISA) results suggested that the levels of IFN‐γ, transforming growth factor β (TGFβ) and TNF‐α in cell culture medium from the DC‐CIK‐OCSC group were significantly higher than those in the OCSC group, the simple CIK‐OCSC group and the DC‐OCSC group (Figure 4). The IL‐2 secretion level in the DC‐CIK‐OCSC group was significantly higher than that in any other group (Figure 4). The IL‐12 secretion levels in the DC‐CIK‐OCSC group and the DC‐OCSC group were significantly higher than that in the simple OCSC group (Figure 4). The IL‐18 secretion levels by immune cells in the 3 co‐culture groups were all significantly higher than that in the OCSC group (Figure 4). The experimental results indicated that DC‐CIK co‐culture resulted in inflammatory factor secretion.

### 3.4 OCPMB‐derived DC‐CIK cells efficiently activated the ASK1‐AIP1‐JNK pathway in OCSCs

**Figure 5:**
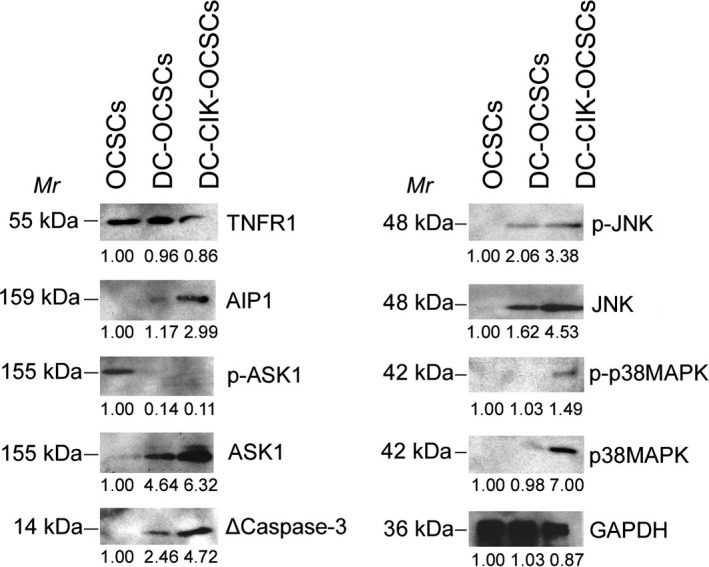
OCPMB‐derived DC‐CIK cells induced high expression of the TNFR1‐ASK1‐AIP1‐JNK pathway. DC and DC‐CIK cells were separately mixed with OCSCs at a ratio of 10:1 and cultured in vitro for 24 h. The Western blot results showed that the expression levels of Ask1, AIP1, JNK, p‐JNK, p38MAPK, p‐p38MAPK and activated caspase‐3 in the DC‐CIK‐OCSC group were significantly higher than those in the DC‐OCSC group and the simple OCSC group

The qPCR results showed that after cells in all groups were co‐cultured for 24 hours, the mRNA expression levels of Tnfr1, Ask1, Aip1, Jnk and caspase‐3 in OCSCs in the DC‐CIK‐OCSC group and the DC‐OCSC group were significantly higher than those in the simple OCSC group (Figure 4). In addition, the mRNA expression levels of the above genes in the DC‐CIK‐OCSC group were significantly higher than those in the DC‐OCSC group (Figure 4), and the expression level of the nuclear proliferation factor mKi67 in the DC‐CIK‐OCSC group was significantly lower than those in the DC‐OCSC group and the simple OCSC group (Figure 4). The Western blot results showed that the expression levels of Ask1, AIP1, JNK, p‐JNK, p38MAPK, p‐p38MAPK and activated caspase‐3 in the DC‐CIK‐OCSC group were significantly higher than those in the DC‐OCSC group and the simple OCSC group (Figure 5); however, analysis of p‐ASK1 expression showed the opposite trend (Figure 5). The experimental results indicated that DC‐CIK cells efficiently activated the ASK1‐AIP1‐JNK pathway in OCSCs.

### 3.5 OCSCs with TNFR1 knockout were resistant to OCPMB‐derived DC‐CIK cells

**Figure 6:**
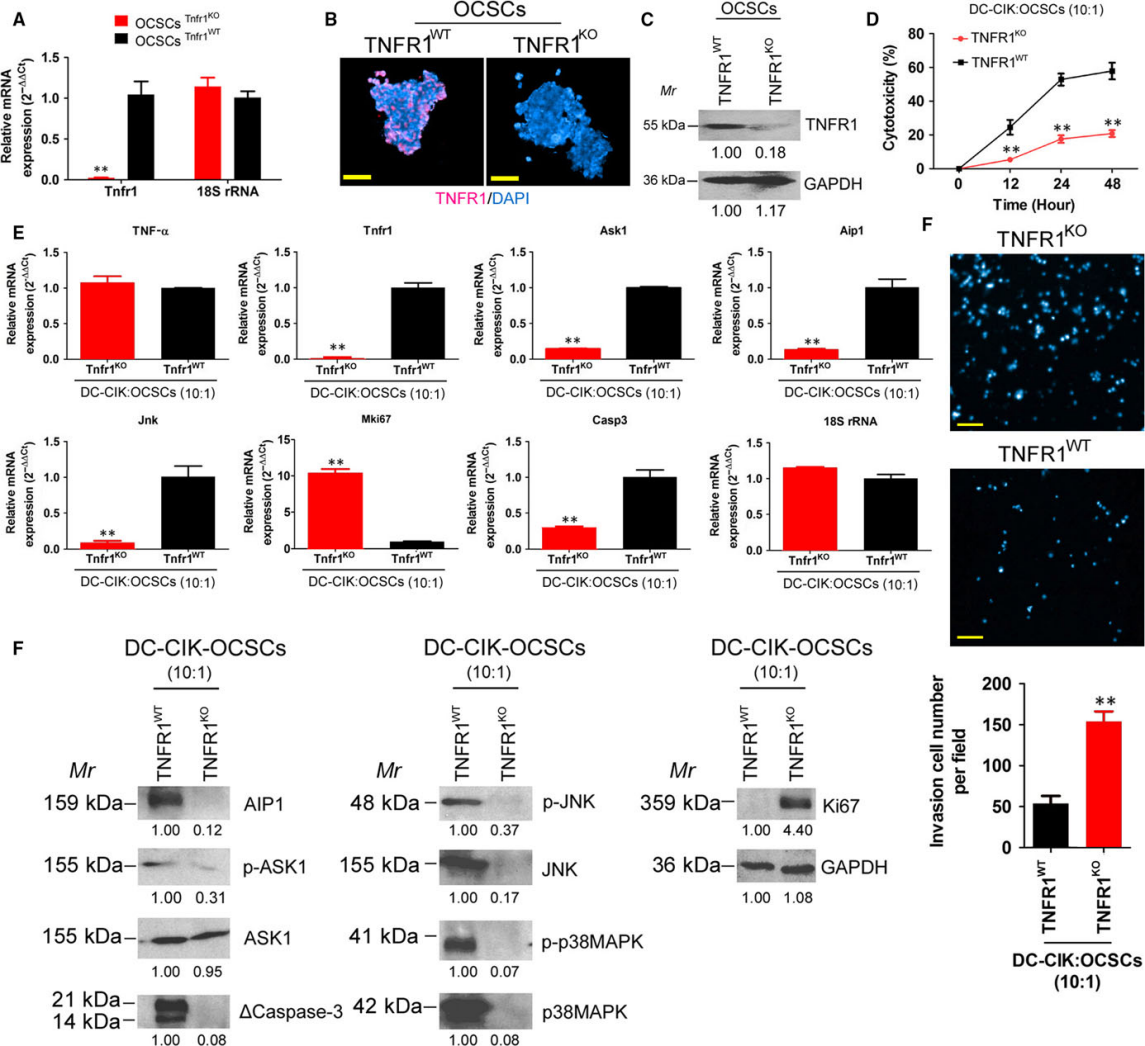
TNFR1 knockout OCSCs were resistant to OCPMB‐derived DC‐CIK cells. A, The qPCR results showed that the expression level of endogenous TNFR1 in the OCSC group transfected with the human TNFR1 CRISPR/Cas9 KO plasmid was significantly lower than that in the group transfected with an empty plasmid. **P < .01 vs OCSCs Tnfr1 WT; n = 4; B, Immunofluorescence staining results showed that OCSCs in the TNFR1KO group barely expressed any TNFR1 protein. Scale bar = 30 μm; C, The Western blot results showed that the expression of endogenous TNFR1 protein in the OCSCs from the TNFR1KO group was significantly lower than that in the TNFR1WT group; D, The cytotoxicity experiment results showed that the cytotoxicity of DC‐CIK cells on OCSCs in the TNFR1KO group was significantly lower than that in the control group. **P < .01 vs OCSCs Tnfr1 WT; n = 4; E, The qPCR results showed that the mRNA expression levels of Tnfra, Ask1, Aip1, Jnk and caspase‐3 in OCSCs in the DC‐CIK‐TNFR1KO group were significantly lower than that in the DC‐CIK‐TNFR1WT group; F, The transwell experiment results showed that the number of migrated OCSCs in the DC‐CIK‐TNFR1KO group was significantly higher than that in the control cell group. **P < .01 vs OCSCs Tnfr1 WT; n = 4. Scale bar = 30 μm; G, Western blot results showed that the expression levels of AIP1, p‐ASK1, JNK, p‐JNK, p38MAPK, p‐p38MAPK and activated caspase‐3 in OCSCs in the DC‐CIK‐TNFR1KO group were significantly lower than those in the DC‐CIK‐TNFR1WT group. **P < .01 vs OCSCs Tnfr1WT; n = 4

**Figure 7:**
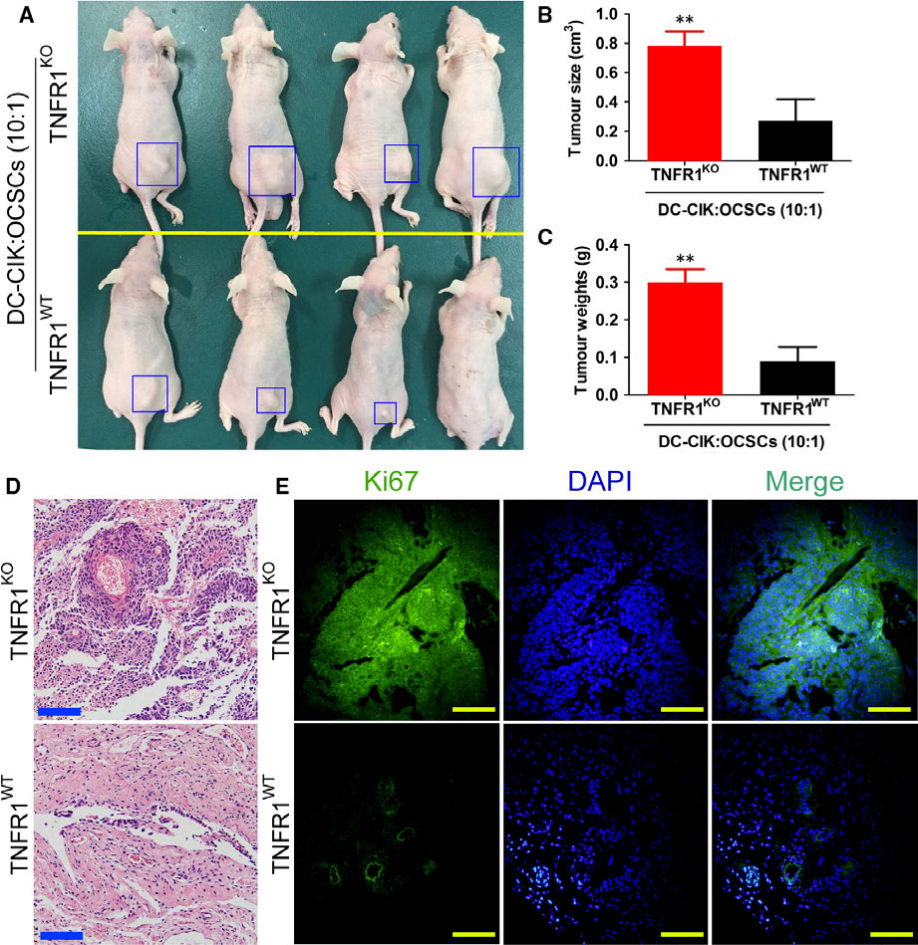
TNFR1 knockout OCSCs readily formed tumours in nude mice. A, The in vivo tumorigenicity experiments in nude mice showed that the visual sizes of tumours in nude mice that developed from OCSC in the DC‐CIK‐TNFR1KO group were significantly larger than those that developed from the DC‐CIK‐TNFR1WT group; B, The volumes of tumours that developed from OCSCs in the DC‐CIK‐TNFR1KO group were significantly larger than those that developed from the DC‐CIK‐TNFR1WT group. **P < .01 vs OCSCs Tnfr1 WT; n = 4; C, The weights of tumours that developed from OCSCs in the DC‐CIK‐TNFR1KO group were significantly higher than those that developed from the DC‐CIK‐TNFR1WT group. **P < .01 vs OCSCs Tnfr1 WT; n = 4; D, Pathological analysis showed that tumours that developed from these 2 groups of cells were all mixed‐type epithelial ovarian cancer. Scale bar = 30 μm; E, The immunofluorescence staining results showed that the expression of ki67 protein in tumours that developed from OCSCs in the DC‐CIK‐TNFR1KO group was higher than that in tumours that developed from the DC‐CIK‐TNFR1WT group. Scale bar = 30 μm

Endogenous TNFR1 expression in OCSCs was knocked out using CRISPR/Cas9 technology. The detection results showed that the expression level of endogenous TNFR1 in the OCSC group transfected with the human TNFR1 CRISPR/Cas9 KO plasmid was significantly lower than that in the group transfected with an empty plasmid (Figure 6). The cytotoxicity experiment results showed that the cytotoxicity of DC‐CIK on OCSCs in the TNFR1KO group was significantly lower than that in the control cell group (Figure 6). The qPCR results showed that the mRNA expression levels of Tnfra, Ask1, Aip1, Jnk and caspase‐3 in OCSCs in the DC‐CIK‐TNFR1KO group were significantly lower than those in the DC‐CIK‐TNFR1WT group (Figure 6). The Western blot results showed that the expression levels of AIP1, p‐ASK1, JNK, p‐JNK, p38MAPK, p‐p38MAPK and activated caspase‐3 in OCSCs in the DC‐CIK‐TNFR1KO group were significantly lower than those in the DC‐CIK‐TNFR1WT group (Figure 6). In addition, the transwell experiment results showed that the number of migrated OCSCs in the DC‐CIK‐TNFR1KO group was significantly higher than that in the control cell group (Figure 6). Furthermore, the results of in vivo tumorigenicity testing in nude mice showed that the size and weight of tumours developed from OCSCs in nude mice in the DC‐CIK‐TNFR1KO group were both significantly higher than those in the control group (Figure 7). The pathological analysis results showed that although the tumours that developed from both cell types in these 2 groups were mixed‐type epithelial ovarian cancer, the tumour tissues that developed from OCSCs in the DC‐CIK‐TNFR1KO group expressed high levels of Ki67 protein (Figure 7). The above results indicated that after endogenous TNFR1 expression in OCSCs was knocked out, cellular sensitivity to DC‐CIK cells was significantly decreased, suggesting that the killing function of DC‐CIK cells was exerted through the TNFR1 receptor.

### 3.6 The TNFR1 expression level negatively correlated with ovarian cancer stage and prognosis

**Figure 8:**
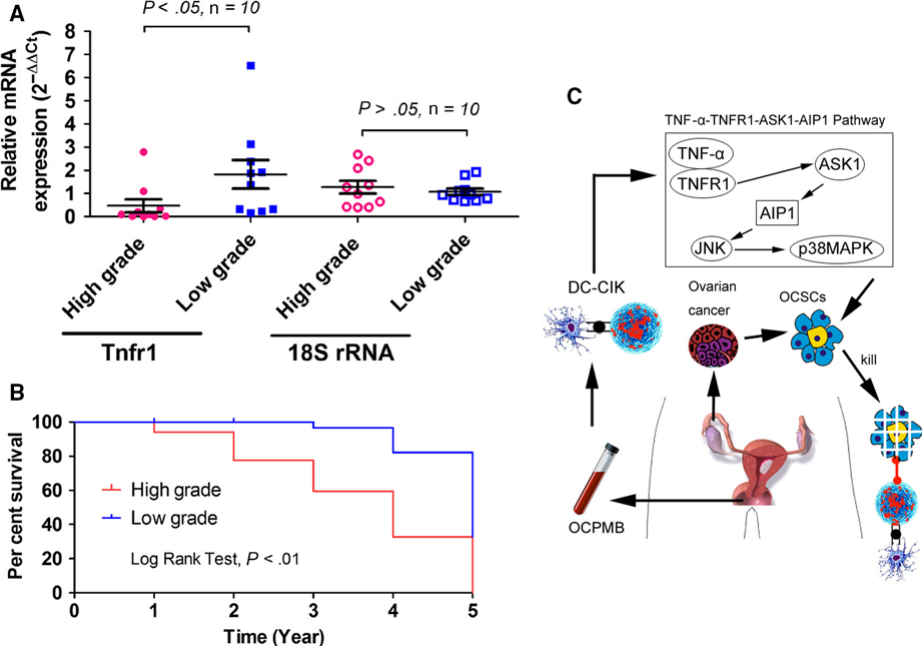
The TNFR1 expression level negatively correlated with ovarian cancer stage and prognosis. A, The qPCR results showed that TNFR1 expression in low‐grade ovarian cancer tissues was higher than that in high‐grade ovarian cancer tissues, and this difference was statistically significant; B, The survival time of ovarian cancer patients with high TNFR1 expression was significantly longer than those with low TNFR1 expression; C, OCPMB‐derived DC‐CIK cells activated the TNFR1‐ASK1‐AIP1‐JNK pathway to kill autologous OCSCs through the secretion of TNF‐α

To explore the relationship between the TNFR1 expression level and ovarian cancer stage and prognosis, tissue samples from 10 cases of high‐grade and 10 cases of low‐grade ovarian cancer patients were collected. The qPCR detection results showed that TNFR1 expression in low‐grade ovarian cancer tissues was significantly higher than that in high‐grade ovarian cancer tissues, and this difference was statistically significant (Figure 8). In addition, analyses of prognosis showed that the survival time of ovarian cancer patients with high TNFR1 expression was significantly longer than that of those with low TNFR1 expression (Figure 8). The experimental results indicated that the TNFR1 expression level negatively correlated with ovarian cancer stage and prognosis.

## 4 DISCUSSION

Ovarian cancer stem cells (mainly the CD44+/CD133+ or the CD44+/CD117+ subpopulations) were discovered in ovarian cancer tissues in recent years; therefore, there are new demands for targeted ovarian cancer therapy.^1^, ^4^ Because OCSCs are resistant to various tumour chemotherapeutic drugs, targeted killing of this cell population is difficult.^1^, ^4^ However, on the other hand, the discovery of one type of tumour treatment method that has high specificity for targeting OCSCs will be very meaningful for the inhibition of ovarian cancer metastasis and recurrence. We investigated CIK cells in this study because we speculated that OCSCs might not be able to tolerate attack by this cell population. Many studies have shown that the drug resistance of OCSCs is mainly present in the timely exclusion of small‐molecule chemotherapeutic drugs outside of cells; therefore, these drugs cannot exert their chemical toxicities.^1^, ^4^The mechanism underlying tumour cell attack by CIK cells is different from that of chemotherapeutic drugs. Therefore, to increase the targeting, we chose other populations of immune cells to assist in cell killing. DCs are the strongest professional APCs in organisms. This population of cells can efficiently uptake, process and present antigens.^5^, ^9^ Mature DCs can present tumour antigens through routes such as MHC‐II to effectively inhibit tumour cell immune escape.^5^, ^9^Many reports have indicated that an effective combination of DC‐CIK cells can exhibit the common high‐efficient killing functions that are present in many types of tumours.^5^, ^9^ In the DC‐CIK treatment, DCs act like “radar” that can recognise antigens to activate the immune response, and CIK cells are similar to a “missile” that can exert cytotoxicity and secret cytokines to accurately kill tumour cells. Therefore, adoptive immunotherapy of malignant tumours using DCs in combination with CIK cells is an emerging and high‐efficient strategy that can effectively control and kill tumour cells without causing obvious side effects, reduce the risk of tumour recurrence and metastasis and increase the immunity and quality of life of patients. Although the DC‐CIK cell therapy has obvious advantages, no reports have demonstrated that the DC‐CIK cell therapy method has obvious killing effects on cancer stem cells (especially OCSCs). Next, allogeneic transplantation of DC‐CIK cells still presents a very high immune rejection risk. The above uncertainties also greatly limit the application of this method in ovarian cancer treatment. Currently, DC‐CIK cells are mainly acquired through venepuncture in patients. However, recurrent punctures for peripheral blood collection present significant trauma and increase the risk of secondary infection in patients with low immunity. Our group analysed cell surface markers to discover that many types of functional cells/stem cells were present in menstrual blood; in addition, transplantation of menstrual blood‐derived endometrial mesenchymal stem cells into mice with premature ovarian failure could achieve the treatment effect.^22^ Furthermore, allogeneic transplantation of this type of cell resulted in very little immune rejection in recipient mice.^21^, ^25^ We hypothesised that if mononuclear cells could be isolated from OCPMB and induced into DC‐CIK cells in vitro, then many deficiencies could be solved. On the one hand, this type of cell is derived from ovarian cancer patients; if they are used for autologous transfusion therapy, the risk of immune rejection will be greatly reduced. On the other hand, menstrual blood discharge is a normal female periodic physiological phenomenon, and the total amount of blood in one discharge is approximately 30‐50 mL; therefore, menstrual blood collection is easy and non‐invasive. If DC‐CIK cells can be successfully obtained from OCPMB, this method will greatly simplify the issue of DC‐CIK sourcing and increase the safety of this approach.

In this study, we referenced some classical DC‐CIK induction methods and changed the seed cells from PBMCs to PBMCs in menstrual blood. In addition, we isolated OSCSs from surgically resected tumour tissues from ovarian cancer patients for in vitro culture. The experimental results indicated that menstrual blood‐derived PBMCs could also be induced using the in vitro cytokine induction method to produce DC‐CIK cells with high efficiency. In addition, further experiments showed that combinations of different ratios of DC‐CIK cells and OCSCs had significant effects on the killing function of DC‐CIK cells. Menstrual blood‐derived DC‐CIK cells also exhibited very strong killing functions in vitro. The DC‐CIK cells effectively inhibited OCSC proliferation and invasion in vitro as well as OCSC tumorigenicity in mice. Therefore, it was confirmed that menstrual blood‐derived DC‐CIK cells had very strong tumour‐killing abilities similar to those of peripheral blood‐derived DC‐CIK cells. Next, DC‐CIK cells could be used for targeted killing of OCSCs. However, we found a very strange phenomenon in this study in that our isolated OCSCs expressed high levels of TNFR1 protein on their cell surfaces. It has been reported that one pathway for DC‐CIK cells to kill tumour cells is to secrete some tumour‐killing factors such as TNF‐α and IFN‐γ. These cytokines can bind to TNFR1 on the surfaces of tumour cells to activate the necrosis or apoptosis pathways in tumour cells and induce tumour cell death.^5^, ^7^ The TNF‐α‐ and TNFR1‐associated TNFR1‐ASK1‐AIP1‐JNK pathway caught our attention.^16^, ^20^, ^26^ The study of Dyari et al^20^ showed that stimulation of the TNF receptor‐1/ASK1/JNK pathway in ovarian cancer using a ω‐3 17,18‐epoxy‐eicosatetraenoic acid analogue significantly promoted cancer cell apoptosis. Zhang et al showed that the 14‐3‐3 protein was an inhibitory factor of ASK1. AIP1 expression could mediate the dissociation of ASK1 from the 14‐3‐3 protein to promote its activation effects following stimulation with TNF‐α.^19^, ^27^ We also analysed the levels of cytokines released into the cell culture supernatant during the in vitro culture of menstrual blood‐derived DC‐CIK cells. The experimental results suggested that the expression levels of factors such as TNF‐α and IFN‐γ were very high. Surprisingly, we found that primary OCSCs derived from ovarian patients expressed very high levels of TNFR1 on the cell surface, and this discovery undoubtedly provided a mechanism for DC‐CIK cells to kill OCSCs. To confirm that TNFR1 expression was the key to OCSC killing by DC‐CIK cells, TNFR1 expression in OCSCs was specifically knocked out using CRISPR/Cas9 technology. After TNFR1 expression was knocked out in OCSCs, the killing effect of DC‐CIK cells significantly decreased, and the proliferation, invasion and tumorigenic abilities of OCSCs significantly increased. Although DC‐CIK cells in all groups secreted higher levels of factors such as TNF‐α and IFN‐γ, the relevant cell death pathways could not be activated when TNFR1 expression was lacking, thus resulting in significant attenuation of DC‐CIK cell‐mediated killing. Therefore, we considered that the expression of TNFR1 had great influence on the effect of DC‐CIK immunotherapy.

Bye the way, there are many advantages of DC‐CIK amplified from menstrual blood when compared with PBMC from peripheral blood. First, for women, the source of menstrual blood is stable. An adult female will exclude more than 20 mL of menstrual blood every month. In view of our study, we found that the mononuclear cells contained in above menstrual blood are sufficient to induce differentiation into DC‐CIK cells in vitro for the treatment of tumours. Therefore, for adult women, menstrual blood collection and DC‐CIK cells induction could be carried out weekly, so that a large number of DC‐CIK cells could be obtained. Secondly, the method of menstrual blood collecting is very simple and safe. Usually, the venous puncture must be carried out to collect the peripheral blood. However, the collection of menstrual blood does not require invasive puncture behaviour. The collection of menstrual blood only needs to put the sterile disposable menstrual cup into the vagina. This operation is simple and safe. Any women who have the ability to take care of themselves can do their own menstrual blood collection. As the collection of menstrual blood does not need to be venepuncture, the risk of infection is greatly reduced.

In summary, this study proposes a new route for acquiring DC‐CIK cells using PBMCs collected from OCPMB to prepare high‐efficient DC‐CIK cells. In addition, this study also provides clues for evaluating the efficacy of DC‐CIK cells; when DC‐CIK immunotherapy is required, whether the TNFR1 molecule is expressed on the surface of cancer cells should be assessed beforehand.

## CONFLICT OF INTEREST

The authors declare that they have no competing interests.
