# Supplementary material for: DC‐CIK cells derived from ovarian cancer patient menstrual blood activate the TNFR1‐ASK1‐AIP1 pathway to kill autologous ovarian cancer stem cells
Source: J Cell Mol Med. 2018 Mar 22;22(7):3364–76. doi: 10.1111/jcmm.13611 (PMC6010766; doi:10.1111/jcmm.13611)
Supplement: Supplementary file 1 [file JCMM-22-3364-s001.doc]

**Table S**1 Characteristics of the patients’ cohort

|  | **Patients (n=6)** |
| --- | --- |
| **Age median (range)**  ≤50  35-48  ≥30 | 32-49 |
|  | 1 |
|  | 3 |
|  | 2 |
| **Surgical staging**  I a, b, c  II a, b, c  III a, b, c  IV |  |
|  | 2 |
|  | 4 |
|  | 0 |
|  | 0 |
| **Histopathology**  Serous  Endometrioid  Mucinous  Clear cells  Mixed epithelial |  |
|  | 1 |
|  | 0 |
|  | 0 |
|  | 1 |
|  | 4 |
| **Tumor Grade**  1  2  3 or clear cell  Unknown |  |
|  | 0 |
|  | 2 |
|  | 4 |
|  | 0 |
| **Treatments**  Primary surgery  Radical surgery  Secondary surgery  Platinum-based chemotherapy  Radiotherapy |  |
|  | 6 |
|  | 0 |
|  | 0 |
|  | 0 |
|  | 0 |
