# Supplementary material for: DC‐CIK cells derived from ovarian cancer patient menstrual blood activate the TNFR1‐ASK1‐AIP1 pathway to kill autologous ovarian cancer stem cells
Source: J Cell Mol Med. 2018 Mar 22;22(7):3364–76. doi: 10.1111/jcmm.13611 (PMC6010766; doi:10.1111/jcmm.13611)
Supplement: Supplementary file 2 [file JCMM-22-3364-s002.doc]

**Table S2** The PCR primer sequences.

| Gene product | Forward (F) and reverse (R) primers (5'→3') | Size (bp) |
| --- | --- | --- |
| Tnfα | F: TCAGCCTCTTCTCCTTCCTG | 124 |
|  | R: GCCAGAGGGCTGATTAGAGA |  |
| Tnfr1 | F: GAGAGGCCATAGCTGTCTGG | 142 |
|  | R: CTCTCTTCTCCCTGTCCCCT |  |
| Ask1 | F: TCGACTTTGGAGAAACCACC | 108 |
|  | R: CCCCAAGGTGGTAAAACAAG |  |
| Aip1 | F: GGCTCAAGTGTGTCAAGCAA | 94 |
|  | R: AGCTCATGGTCCACAGAACC |  |
| Jnk | F: CAAGCACCTTCATTCTGCTG | 102 |
|  | R: GCCAGACCGAAGTCAAGAAT |  |
| Mki67 | F: CCTGTACGGCTAAAACATGGA | 128 |
|  | R: GCTGGCTCCTGTTCACGTA |  |
| Casp3 (Caspase 3) | F: CTGCCTCTTCCCCCATTCT | 110 |
|  | R: TCGCTTCCATGTATGATCTTTG |  |
| 18s rRNA | F: CGTTGATTAAGTCCCTGCCCTT | 202 |
|  | R: TCAAGTTCGACCGTCTTCTCAG |  |
