# Supplementary material for: DC‐CIK cells derived from ovarian cancer patient menstrual blood activate the TNFR1‐ASK1‐AIP1 pathway to kill autologous ovarian cancer stem cells
Source: J Cell Mol Med. 2018 Mar 22;22(7):3364–76. doi: 10.1111/jcmm.13611 (PMC6010766; doi:10.1111/jcmm.13611)
Supplement: Supplementary file 3 [file JCMM-22-3364-s003.docx]

**Table S3 Antibodies**

| Catalog no. | Name | Application and Dilution | Company |
| --- | --- | --- | --- |
| ab8348 | Mouse anti-Human TNF alpha Ab | WB (1:1000)  IF (1:300) | Abcam (Cambridge, MA, USA) |
| ab19139 | Rabbit anti-human TNF Receptor I Ab | WB (1:1000) | Abcam (Cambridge, MA, USA) |
| ab45178 | Rabbit anti-human ASK1 Ab | WB (1:1000)  IF (1:300) | Abcam (Cambridge, MA, USA) |
| ab47304 | Rabbit anti-human ASK1 (phospho S83) Ab | WB (1:1000) | Abcam (Cambridge, MA, USA) |
| ab181603 | Rabbit anti-human GAPDH Ab | WB (1:1000) | Abcam (Cambridge, MA, USA) |
| ab209674 | Rabbit anti-human AIP1 Ab | WB (1:1000) | Abcam (Cambridge, MA, USA) |
| ab170099 | Rabbit anti-human p38 Ab | WB (1:1000) | Abcam (Cambridge, MA, USA) |
| ab47363 | Rabbit anti-human p38 (phospho Y182) Ab | WB (1:1000) | Abcam (Cambridge, MA, USA) |
| ab208035 | Rabbit anti-human JNK1+JNK2+JNK3 Ab | WB (1:1000) | Abcam (Cambridge, MA, USA) |
| ab124956 | Rabbit anti-human JNK1 + JNK2 + JNK3 (phospho T183+T183+T221) Ab | WB (1:1000) | Abcam (Cambridge, MA, USA) |
| ab15580 | Rabbit anti-human Ki67 Ab | WB (1:1000)  IF (1:300) | Abcam (Cambridge, MA, USA) |
| ab13585 | Mouse anti-human Caspase 3 Ab | WB (1:1000) | Abcam (Cambridge, MA, USA) |
| ab6785 | Goat Anti-Mouse IgG H&L (FITC) Ab | IF (1:300) | Abcam (Cambridge, MA, USA) |
| ab6717 | Goat Anti-Rabbit IgG H&L (FITC) Ab | IF (1:300) | Abcam (Cambridge, MA, USA) |
| ab6789 | Goat Anti-Rabbit IgG H&L (HRP) | WB (1:1000) | Abcam (Cambridge, MA, USA) |
| ab6728 | Goat Anti-Mouse IgG H&L (HRP) | WB (1:1000) | Abcam (Cambridge, MA, USA) |
